# A nurse-delivered, clinic-based intervention to address intimate partner violence among low-income women in Mexico City: findings from a cluster randomized controlled trial

**DOI:** 10.1186/s12916-017-0880-y

**Published:** 2017-07-12

**Authors:** Jhumka Gupta, Kathryn L. Falb, Oriana Ponta, Ziming Xuan, Paola Abril Campos, Annabel Arellano Gomez, Jimena Valades, Gisele Cariño, Claudia Diaz Olavarrieta

**Affiliations:** 1 Department of Global and Community Health, George Mason University, MS 5B7, 4400 University Drive, Fairfax, VA 22030 USA; 2 International Rescue Committee, 122 East 42nd Street, New York, NY 10168 USA; 3 Innovations for Poverty Action, Manuel María Contreras 133, Mezzanine 2 Col. Cuauhtemoc, Mexico City, 06500 Mexico; 4 Department of Community Health Sciences, Boston University School of Public Health, 801 Massachusetts Avenue, Boston, MA 02118 USA; 5 Mexico City Ministry of Health, Xocongo # 225, Col. Transito, Mexico City, 068020 Mexico; 6 International Planned Parenthood Federation, 125 Maiden Lane, New York, NY 10038 USA; 7 Population Council, Av. Insurgentes Sur No. 2453 Torre Murano, Piso 9, Local 903, Col. Tizapán, Delegación Álvaro Obregón, Mexico City, 01090 Mexico

**Keywords:** Intimate partner violence, Violence against women, Randomized controlled trial, Screening, Safety planning, Health sector, Latin America and the Caribbean

## Abstract

**Background:**

Rigorous evaluations of health sector interventions addressing intimate partner violence (IPV) in low- and middle-income countries are lacking. We aimed to assess whether an enhanced nurse-delivered intervention would reduce IPV and improve levels of safety planning behaviors, use of community resources, reproductive coercion, and mental quality of life.

**Methods:**

We randomized 42 public health clinics in Mexico City to treatment or control arms. In treatment clinics, women received the nurse-delivered session (IPV screening, supportive referrals, health/safety risk assessments) at baseline (T1), and a booster counselling session after 3 months (T2). In control clinics, women received screening and a referral card from nurses. Surveys were conducted at T1, T2, and T3 (15 months from baseline). Our main outcome was past-year physical and sexual IPV. Intent-to-treat analyses were conducted via three-level random intercepts models to evaluate the interaction term for treatment status by time.

**Results:**

Between April and October 2013, 950 women (480 in control clinics, 470 in treatment clinics) with recent IPV experiences enrolled in the study. While reductions in IPV were observed for both women enrolled in treatment (OR, 0.40; 95% CI, 0.28–0.55; *P* < 0.01) and control (OR, 0.51; 95% CI, 0.36–0.72; *P* < 0.01) clinics at T3 (July to December 2014), no significant treatment effects were observed (OR, 0.78; 95% CI, 0.49–1.24; *P* = 0.30). At T2 (July to December 2013), women in treatment clinics reported significant improvements, compared to women in control clinics, in mental quality of life (β, 1.45; 95% CI, 0.14–2.75; *P* = 0.03) and safety planning behaviors (β, 0.41; 95% CI, 0.02–0.79; *P* = 0.04).

**Conclusion:**

While reductions in IPV levels were seen among women in both treatment and control clinics, the enhanced nurse intervention was no more effective in reducing IPV. The enhanced nursing intervention may offer short-term improvements in addressing safety planning and mental quality of life. Nurses can play a supportive role in assisting women with IPV experiences.

**Trial Registration:**

Clinicaltrials.gov (NCT01661504). Registration Date: August 2, 2012

**Electronic supplementary material:**

The online version of this article (doi:10.1186/s12916-017-0880-y) contains supplementary material, which is available to authorized users.

## Background

The harmful public health impacts of male-perpetrated intimate partner violence (IPV) against women have been extensively documented, and include increased vulnerability to poor mental health, HIV, sexual and reproductive health problems, injury, and death [1, 2]. Globally, one in three women report experiencing such violence, with greater prevalence found in low- and middle-income countries (LMICs) [1].

The healthcare sector has long been highlighted for its critical role in combating IPV. This is due in part to the high prevalence of IPV among women who seek health services and because healthcare providers have access to this otherwise isolated population [3, 4]. However, sufficient evidence does not exist to guide healthcare providers on how to most effectively meet the needs of such women. A review of existing data from randomized trials on routine screening of all women for IPV experiences by healthcare providers indicated that reductions in IPV and improvements in other health behaviors and outcomes have not been consistent [5]. As such, even though the World Health Organization provides clinical guidelines for addressing IPV within primary care [6], it does not support routine screening for IPV. Common approaches within primary care settings include healthcare provider delivered education and counselling regarding IPV and related health concerns [7]. To date, however, healthcare sector interventions within primary care settings that focus on intervening with women identified for IPV through screening have also yielded mixed or sub-optimal findings regarding improvements in mental health, reduced experiences of IPV, and help seeking and safety planning behaviors [8–11].

Within LMICs, rigorous evaluations of health sector interventions to address IPV are particularly scarce. Numerous challenges exist in these contexts to implementing health sector IPV programming and in conducting rigorous evaluation trials that include overburdened health facilities, high staff turnover, lack of private spaces within healthcare facilities, victim-blaming attitudes held by providers, and weak referral networks [3]. Public (i.e., government run) facilities may face additional challenges with the follow-up of women since they may serve a lower-income and more vulnerable portion of the population than privately run healthcare facilities [12]. Moreover, as shown in a Mexico-based study [13], nursing staff may experience IPV at a level similar to the general population, which may pose personal obstacles to an IPV response due to normalization of such behavior. Regardless of such challenges, there is increasing evidence of high-level commitment to addressing IPV within governments, as Mexico ratified the Convention to End All forms of Discrimination Against Women and Children in 1981 and the Optional Protocol 2002 [14], and in 2007, the Mexican government passed the General Law on Women’s Access to a Life Free From Violence [15]. The implementation of clinic-based IPV programming in LMICs is also increasingly being documented, and their governments are also being called upon to increase the prioritization of IPV within health sector budgets and policies [3, 7, 16]. A recent review conducted by the Pan American World Health Organization of clinical guidelines for responding to IPV in 12 out of 18 Latin American and Caribbean countries also underscored the importance of increased training of healthcare providers [17]. To optimally inform such efforts, data from rigorous evaluations of health sector responses to IPV within LMICs are urgently needed.

The current study aimed to address this important gap in the intervention literature through conducting a clinic-based randomized controlled trial (RCT) to assess the impact of a nurse-delivered intervention to women identified as experiencing recent IPV through screening within public health clinics in Mexico City. The intervention aimed to reduce IPV, improve use of community resources, increase safety planning, decrease reproductive coercion, and improve mental quality of life more so than the provision of resources alone.

## Methods

### Study design

This study is a cluster-randomized controlled trial that occurred between 2012 and 2015 across 42 public health clinics operated by the Ministry of Health (MoH) in Mexico City. A full study protocol is described elsewhere [12]. Briefly, in April 2013, the study began a rolling recruitment and baseline survey administration (Time 1; T1). Women were then invited to participate in a 3-month follow-up survey (Time 2; T2) and a 15-month follow-up survey (Time 3; T3). The 15-month follow-up period was selected to allow for the research team to assess for past-year changes in IPV between T2 and T3. All survey data were collected through the Computer Assisted Self-Interview on laptops, where trained research assistants read each item and response choices aloud to participants, and entered the response choice on participants’ behalf. This approach, versus audio computer assisted interviewing was implemented based on piloting, where participants indicated greater comfort with the research assistant-assisted interview. Interviews were completed in private areas of the clinics and research assistants were available for questions throughout the surveys.

The T2 survey was conducted before the booster counseling session and women received US$15 for their time in the form of a gift card. At T3, women received US$20 in the form of a gift card.

At all data collection points, women were asked to provide informed consent. Specifically, in a private location within the clinic, trained research assistants told eligible women that they were invited to take part in a research study to learn about women’s health, family, and relationships, including difficult issues like violence. They were told that they would be invited to take part in three surveys in total (with time points specified), that they could withdraw at any time and that their responses would be kept private, and that their decisions to participate or not would not impact the care they receive at the health clinic. They were also told that the clinic they were visiting was part of a group of clinics in Mexico City that were participating in the study, and that for the purpose of the study, clinics were randomly assigned to offer one of two types of services: comprehensive services to assist women with health and family issues or a shorter version of the services. Women were told that neither the research team nor the nurse would be able to share whether their clinic would be offering comprehensive services or the shorter version.

Mexico-based research staff (all of whom had at least an undergraduate degree) were also trained on research and ethical protocols that adhered to international standards on violence against women research, including intervention research [18, 19], and were approved by the Yale School of Public Health (#1202007993), George Mason University (protocol # 704016–4), Innovations for Poverty Action (#00006083), and the Mexico City MoH (protocol #1470-6812) human subjects committees.

### Participants

**Fig. 1:**
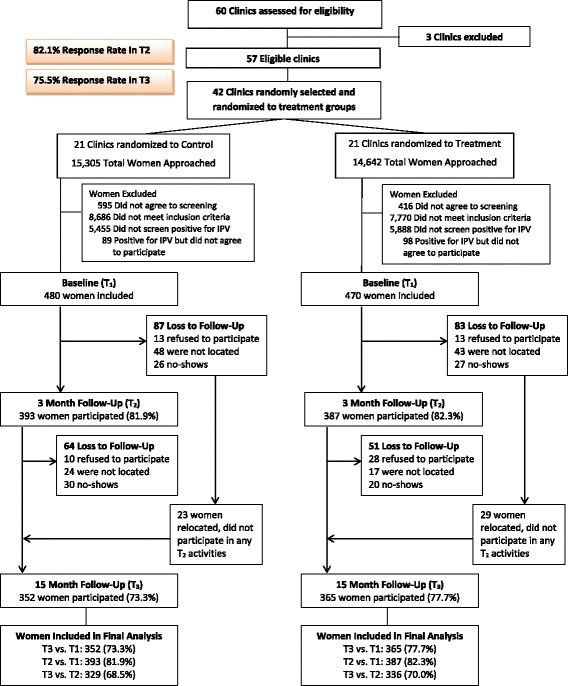
Consort diagram

A total of 29,947 women presenting at the participating 42 clinics (details of clinic selection are described in the “randomization and masking” section) were approached and screened for eligibility (see Consort Diagram, Fig. 1). Criteria for participation included being between 18 and 44 years of age, currently in a heterosexual relationship, reporting experiences of physical and/or sexual IPV within the past year in research assistant-administered screening, and were not pregnant or were pregnant in their first trimester. Based on feedback from piloting the assessment, the IPV screening tool consisted of 11 questions in order to build rapport between the research assistant and the participant. The first nine questions were in regards to the woman’s health and relationship with her partner, including emotional abuse, as directly asking about physical or sexual IPV at the very beginning of the assessment was rendered as too sensitive during our piloting phase. Based on feedback from focus groups carried out with IPV survivors at a community domestic violence agency, concrete examples of physical and sexual IPV were included in the questions. More details on this screening tool can be viewed in the study protocol [12]. Exclusion criteria included having plans to relocate in the next 2 years or having an easily recognizable cognitive impairment.

### Randomization and masking

Clinics were eligible to participate if they were Type III clinics, which are larger government-led community health clinics that provide more comprehensive care and services (in comparison to smaller Types I and II clinics). These Type III clinics serve a lower income population within Mexico City. A total of 60 Type III clinics were assessed for eligibility. Three clinics were excluded from the study; two were eliminated to reduce the threat of contamination (i.e., were located in close proximity to a MoH hospital that offered IPV programming) and one was eliminated given that it was located within a small catchment area with few community services. Of these 57 eligible clinics, 42 were randomly selected using excel, based on sample size calculations. Health centers selected were stratified by city zone and borough using an excel file. Health centers were assigned a number using the RAND command to randomly select the health centers [20]. Specifically, to randomly select the 42 health centers, all centers were assigned random numbers in Excel and sorted from smallest to largest; health centers were selected based on city zone and in order of their random number. More details on the sample size and power calculations can be reviewed in a previously published protocol paper [12].

The 42 health centers were stratified by zone and district, and randomly assigned to treatment or control using STATA [21]; more details can be reviewed in the previously published protocol paper [12]. To ensure that the neighborhoods where the clinics were located were similar in terms of degree of urbanization and socioeconomic status, data from the National Institute of Statistics were used to create a socioeconomic index variable based on borough-level aggregate data on levels of education, number of rooms in the house, concrete floor, household items (television, internet, car, refrigerator, computer, and bathrooms). Treatment and control clinics did not differ significantly at the 5% level on these variables. Participants were blinded to their study arm, while nurses, clinic staff, and the research team were not.

### Procedures

The intervention consisted of the following components as delivered by nurses (Table 1): (1) integrated IPV and health screening assessment; (2) supportive care; (3) safety planning and harm reduction counseling, including reproductive health concerns; (4) assisted referrals; and (5) a booster counseling session at 3 months after the initial screening and counseling session. Women at the treatment clinics received the intervention from nurses who were eligible and selected to participate in a 3-day training that covered topics related to IPV, safety planning, reproductive coercion, and community resource referrals, and was developed jointly by the research team and the International Planned Parenthood Federation/Western Hemisphere Region [22]. Nurses from all 42 health clinics were invited to participate in the training based on whether they were morning shift nurses (due to the walk-in basis of appointments and afternoon shifts not occurring at all clinics) and not a field nurse (nurses who work in the community and thus have limited hours at health clinics). Out of 379 eligible nurses, 197 (52.0%) were trained for the study. As part of the training, the research team also conducted an additional three clinic visits to practice via supervised role-playing exercises. Women in control clinics received the MoH standard of care, which consisted of a referral card containing information on IPV and available MoH and community services. Additional details regarding the nurse selection and participation, development, delivery, and components of the intervention can be found in the study protocol [12].

**Table 1:** Components of interventions for treatment and control clinics

Intervention component	Treatment clinics	Control clinics
Integrated IPV and health screening	Women were screened for IPV including emotional, physical, and sexual violence, as part of a general health assessment.	Integrated IPV and health Screening, Supportive Care, Business-sized Referral Card, Booster session at 3 months where the referral card was redistributed.
Supportive care	Nurses were trained to provide non-judgmental and empathetic counseling.	
Safety planning and harm reduction counseling	Nurses discussed safety planning measures with women, including escape routes or places of refuge, packing and storing a bag with important belongings, memorizing phone numbers, talking to children about what to do in cases of violence, and staying away from rooms with weapons. Harm reduction counseling included the partners’ use of alcohol and illicit drugs, how to remove weapons, options for protecting reproductive health, such as protecting against unplanned pregnancy, sexually transmitted infections, and other individual-specific health risks.	
Supportive referrals	Nurses provided information regarding local and free IPV resources, in accordance with their specific needs. Nurses facilitated access and utilization by either contacting programs together, or by providing women with step-by-step directions. For the latter, nurses provided specific names of staff members at programs as opposed to a generic address. This information was also provided via business-sized referral cards.	
Booster counseling sessions at 3 months (T2)	Components of above screening, referral, safety planning and harm reduction were reviewed and redelivered to program participants. Sessions occurred in the clinic during an appointment that was scheduled during T1.	

### Outcomes

The primary outcome was physical and sexual IPV. At T1, the time reference was past-year, T2 referred to only experiences of IPV in the past 3 months, and T3 referred to both past year and past 3 months. Any affirmative response to a binary item in both the physical and sexual IPV scales, as drawn from the World Health Organization Multi-Country Study on Domestic Violence and Women’s Health (see Additional file 1, 2, 3 and 4 for complete list of IPV items) [23], was coded as experiencing physical and sexual IPV. Secondary measures included reproductive coercion, whereby any positive response to an item was coded as experiencing reproductive coercion [24], use of community resources [25], safety planning activities [25], and SF-12 quality of life measure (mental quality of life) [26]. Physical quality of life was not an outcome because we did not expect changes in physical quality of life based on the intervention. All measures were piloted and safety planning measures and quality of life scales were adapted as appropriate. For example, this survey employed a 13-item safety planning scale that removed two original items related to having insurance policies available and social security numbers, which were not relevant for this context. The quality of life scale was also adapted from five-point to four-point Likert scales based on pilot feedback. Transformed composite scores for SF-12 quality of life measure were respectively calculated for mental health scores as a continuous outcome [27].

### Statistical analysis

To account for the clustering in the data structure where baseline and follow-up outcomes were repeated measures nested within individuals, which were nested within health clinics, multilevel analysis was conducted to model changes of IPV and related outcomes by treatment status. Specifically, a three-level random intercepts model was conducted to evaluate the interaction term between treatment status and time (e.g., T1 vs. T3) with the use of random effects to adjust for both correlation between the time points within individuals and clustering among individuals nested within health clinics. We employed the generalized mixed model in GLIMMIX procedure in SAS v9.2 [28] to fit the multilevel model. A significant treatment by time interaction term suggested statistically significant differential effects of treatment on changes in outcomes from T1 to T3. Covariates deemed substantively important based on prior literature or significant between-group differences found at baseline were adjusted in the multiple regression models. Odds ratios (ORs), 95% confidence intervals (CIs), and *P* values were used to assess the significance of the regression coefficients for models with binary outcomes, while β, 95% CIs, and *P* values were used for continuous outcomes. Specifically, we first evaluated whether there was significant change of outcome within each treatment group, then we compared whether such outcome change differed by treatment status. Because the main research question was to investigate the differential treatment effect by group status, β and *P* values were used for evaluating the significance of the treatment by time interaction terms.

To assess nurse fidelity to the intervention protocol, exit interviews with women in both treatment and control clinics were conducted immediately after their meeting with nurses at T1. All participants were approached, and 2.6% (n = 25) of study participants did not complete the exit survey. Of these 25 participants, 16 completed the survey, but left the health center before meeting with the nurse. The remaining nine participants declined participation in the exit interview due to insufficient time. A high adherence to the intervention protocol was observed in both treatment and control exit interviews (treatment mean =15.3 out of 16 possible points; control mean = 5.69 out of 6 possible points).

## Results

The resulting sample size was 950 participants (480 in control clinics; 470 in treatment clinics) at T1 (participation rate of 83.6% among women who could be determined to be eligible). See Fig. 1 for more details.

At T2, 780 women, or 82.1% of the baseline sample, participated in the survey. There was no differential attrition by study arms for T1 demographics nor IPV status at T2. The primary reason for loss to follow-up was not being able to be located by the research team, followed by being a “no-show”, and refusal to participate. In order to reduce loss to follow-up, all women received monthly phone call reminders and home visits for their future appointments [12]. Regardless of whether a woman participated in T2, all women were invited to complete the T3 survey. Of the original sample, 717 women completed the T3 survey, or 75.5% of the baseline sample. Fifty-two women who could not be located during T2 participated in T3. Differential attrition in past month physical and sexual IPV was observed between T2 and T3; control participants who were lost to follow-up from T2 to T3 reported more past month physical and sexual IPV at T2 than treatment participants; no other differences regarding attrition were observed. Overall, 69.3% of women included in the baseline participated in both periods of data collection.

Demographics of participants are presented in Table 2 for the overall sample and by treatment group. No statistically significant demographic differences were found between treatment arms (thus randomization was largely successful), nor were there any statistically significant differences in study outcomes reported at T1. Considering prior literature and the between-group baseline comparisons, we further adjusted for age, number of children, and birthplace in the subsequent multiple regression models.

**Table 2:** Baseline (T1) characteristics of study sample, by treatment arm (*N* = 950)

	Treatment (*n* = 470) Mean (SD) or *n* (%)	Control (*n* = 480) Mean (SD) or *n* (%)	*t* statistic or χ^2^	*P* value
Age, years	30.12 (7.28)	29.60 (7.03)	1.12	0.26
Number of children	2.28 (1.18)	2.13 (1.22)	1.98	0.05
Partner’s age minus woman’s age, years	3.19 (5.90)	3.50 (6.66)	0.78	0.44
Previously screened for IPV in healthcare setting	43 (9.15%)	52 (10.86%)	0.77	0.38
Reason for visit^a^			1.25	0.54
General appointment	108 (22.98%)	124 (25.83%)		
Gynecological appointment	34 (7.23%)	36 (7.50%)		
Other	328 (69.79%)	318 (66.25%)		
Legal status			3.07	0.38
Single	22 (4.68%)	35 (7.29%)		
Married	112 (23.83%)	115 (23.96%)		
Common law marriage	321 (68.30%)	317 (66.04%)		
Separated/Divorced	15 (3.19%)	13 (2.71%)		
Birthplace			1.35	0.25
Mexico City	344 (73.19%)	335 (69.79%)		
State of Mexico	45 (9.57%)	39 (8.13%)		
Another state/country	81 (17.23%)	106 (22.08%)		
Schooling			4.33	0.63
No schooling	16 (3.40%)	10 (2.08%)		
Primary	99 (21.06%)	108 (22.50%)		
Secondary	196 (41.70%)	205 (42.50%)		
High school	100 (21.28%)	86 (17.92%)		
Technical degree	35 (7.45%)	45 (9.38%)		
College	22 (4.68%)	24 (5.00%)		
Post-graduate	2 (0.43%)	2 (0.42%)		
Monthly income MXN (USD)^a^			3.33	0.19
Under $2 K (133)	170 (61.59%)	148 (54.01%)		
$2 K to $4 K (133–266)	86 (30.80%)	99 (36.13%)		
$4 K+ (266+)	21 (7.61%)	27 (9.85%)		
Religion^a^			3.03	0.39
Catholic	385 (81.91%)	385 (80.38%)		
Christian	29 (6.17%)	36 (7.52%)		
None	35 (7.45%)	28 (5.85%)		
Other	21 (4.47%)	30 (6.26%)		
Past-year IPV				
Physical violence	454 (96.60%)	470 (97.92%)	1.56	0.21
Sexual violence	184 (39.15%)	162 (33.89%)	2.83	0.09
Physical and sexual violence	168 (35.74%)	152 (31.67%)	1.77	0.18
Reproductive coercion	106 (34.64%)	112 (34.70%)	0.00	0.97
Safety planning, past 12 months	3.16 (2.85)	3.16 (2.91)	0.02	0.99
Use of community resources, past 6 months	0.30 (0.68)	0.30 (0.78)	0.03	0.97
Quality of life score (mental)	35.14 (7.45)	35.29 (7.90)	0.31	0.76

^a^ *n* values do not total to 950 due to missing values

*IPV* intimate partner violence

Intention-to-treat analysis indicated no significant time by treatment impact for past year IPVs, reproductive coercion, safety planning behaviors, use of community resources, and mental quality of life (Table 3). Both intervention and control participants reported significant reductions in past year IPV (OR, 0.40; 95% CI, 0.28–0.55; *P* < 0.01 and OR, 0.51; 95% CI, 0.36–0.72; *P* < 0.01, respectively), while only treatment participants reported significant reductions in reproductive coercion (OR, 0.56; 95% CI, 0.37–0.83; *P* < 0.01). Both intervention and control participants reported significant increases in safety planning (β, 0.88; 95% CI, 0.58–1.18; *P* < 0.01 and β, 0.52; 95% CI, 0.20–0.83; *P* < 0.01, respectively) and improvement in mental quality of life (β, 2.34; *P* < 0.01 and β, 1.46; *P* < 0.01, respectively), while only women in treatment clinics reported significant improvements in use of community resources (β, 0.20; 95% CI, 0.08–0.31; *P* < 0.01).

**Table 3:** Distribution of study outcomes at T1 and T3, by treatment arm and effect estimates of primary and secondary outcomes (Intent to Treat Analysis)

				T1-T3 Comparison		Treatment X Time interaction	
	Treatment type	Baseline (T1) *N* (%)	Endline (T3) *N* (%)	OR (95% CI)	*p*-value	OR (95% CI)	*p*-value
Physical and Sexual IPV (past year)	Intervention	168/470 (35.7%)	72/365 (19.7%)	0.40 (0.28, 0.55)	<0.01*	0.78 (0.49, 1.24)	0.30
	Control	152/480 (31.7%)	72/352 (20.5%)	0.51 (0.36, 0.72)	<0.01*		
Physical IPV (past year)	Intervention	454/470 (96.6%)	209/365 (57.3%)	0.05 (0.03, 0.08)	<0.01*	1.48 (0.63, 3.49)	0.37
	Control	470/480 (97.9%)	210/351 (59.8%)	0.03 (0.016, 0.06)	<0.01*		
Sexual IPV (past year)	Intervention	184/470 (39.2%)	94/365 (25.8%)	0.47 (0.34, 0.64)	<0.01*	0.90 (0.57, 1.41)	0.65
	Control	162/478 (33.9%)	81/351 (23.1%)	0.54 (0.39, 0.75)	<0.01*		
Reproductive Coercion (past year)	Intervention	106/306 (34.6%)	52/226 (23.0%)	0.56 (0.37, 0.83)	<0.01*	0.71 (0.41, 1.23)	0.22
	Control	112/322 (34.8%)	65/220 (29.6%)	0.79 (0.54, 1.17)	0.23		
	Treatment type	Baseline (T1) Mean (SD)	Endline (T3) Mean (SD)	beta (95% CI)	*p*-value	beta (95% CI)	*p*-value
Use of community resources^a^ (past 6 mos)	Intervention	*N* = 470, 0.30 (0.68)	*N* = 365, 0.51 (1.10)	0.20 (0.08, 0.31)	<0.01*	0.08 (-0.09, 0.24)	0.36
	Control	*N* = 479, 0.30 (0.78)	*N* = 361, 0.43 (1.04)	0.11 (-0.003, 0.23)	0.056		
Safety planning behaviors^a^ (ever T1 vs. past 12 mos T3)	Intervention	*N* = 470, 3.16 (2.85)	*N* = 365, 4.08 (3.34)	0.88 (0.58, 1.18)	<0.01*	0.36 (-0.07, 0.79)	0.10
	Control	*N* = 479, 3.16 (2.91)	*N* = 351, 3.70 (3.24)	0.52 (0.20, 0.83)	<0.01*		
Quality of Life^a^ (mental past month)	Intervention	*N* = 470, 35.14 (7.45)	*N* = 363, 37.20 (7.69)	2.34 (1.41, 3.27)	<0.01*	0.90 (-0.43, 2.24)	0.19
	Control	*N* = 479, 35.39 (7.80)	*N* = 350, 36.42 (7.99)	1.46 (0.48, 2.44)	<0.01*		

^a^ Outcome variable is treated as a continuous variable, therefore regression coefficient beta and 95% confident intervals of beta are reported

* Denotes significant finding

Intention-to-treat analyses found a significant differential increase of safety planning behaviors (β, 0.41; 95% CI, 0.02–0.79; *P* = 0.04) and improvement of mental quality of life (β, 1.45; 95% CI, 0.14–2.75; *P* = 0.03) at T2 among the treatment group relative to the control (Table 4). Both treatment and control participants reported significant increases in use of community resources.

**Table 4:** Distribution of study outcomes at T1 and T2 (3 months), by treatment group and effect estimates of secondary outcomes (intent-to-treat analysis)

				Pre/Post T1–T2 comparison		Treatment × time interaction to examine intervention effects	
	Treatment type	Baseline (T1) *n* Mean (SD)	Midline (T2) *n* Mean (SD)	β (95% CI)	*P* value	β (95% CI)	*P* value
Use of community resources^a^ (ever used measured at T1 vs. 3 months T2)	Intervention	*n* = 470, 0.75 (1.16)	*n* = 387, 0.61 (1.22)	−0.16 (−0.30 to −0.02)	0.02*	0.13 (−0.05 to 0.32)	0.17
	Control	*n* = 479, 0.73 (1.17)	*n* = 393, 0.47 (1.18)	−0.30 (−0.42 to −0.17)	<0.01*		
Safety planning behaviors^a^ (ever done at T1 vs. 3 months T2)	Intervention	*n* = 470, 3.16 (2.85)	*n* = 387, 3.70 (3.11)	0.48 (0.22–0.75)	<0.01*	0.41 (0.02– 0.79)	0.04*
	Control	*n* = 479, 3.16 (2.91)	*n* = 393, 3.31 (3.03)	0.08 (−0.19 to 0.36)	0.56		
Quality of life^a^ (mental, past month)	Intervention	*n* = 470, 35.14 (7.45)	*n* = 386, 37.85 (8.30)	2.85 (1.91–3.79)	<0.01*	1.45 (0.14– 2.75)	0.03*
	Control	*n* = 479, 35.29 (7.90)	*n* = 392, 36.49 (7.63)	1.40 (0.49–2.31)	<0.01*		

^a^Outcome variable is treated as a continuous variable, therefore regression coefficient β and its 95% confidence interval are reported

* Denotes significant finding

*IPV* intimate partner violence

Intention-to-treat analyses found no significant differential effects for IPVs, reproductive coercion, and mental quality of life when comparing T2 and T3 responses (Table 5). Details of the regression coefficients in the final adjusted models with and without the time by treatment interaction are provided in Additional file 1.

**Table 5:** Distribution of study outcomes at T2 and T3, by treatment group and effect estimates of primary and secondary outcomes (Intent to Treat Analysis)

				T2-T3 Comparison		Treatment X Time interaction	
	Treatment type	Midline (T2) *N* (%)	Endline (T3) *N* (%)	OR (95% CI)	*p*-value	OR (95% CI)	*p*-value
Physical and Sexual IPV (past month)	Intervention	33/387 (8.5%)	29/365 (8.0%)	0.84 (0.49, 1.45)	0.53	0.55 (0.26, 1.13)	0.10
	Control	60/393 (15.3%)	29/352 (8.2%)	0.50 (0.31, 0.83)	<0.01*		
Physical IPV (past month)	Intervention	138/386 (35.8%)	91/365 (24.9%)	0.59 (0.43, 0.82)	<0.01*	0.82 (0.52, 1.28)	0.380
	Control	173/393 (44.0%)	97/351 (27.6%)	0.48 (0.35, 0.66)	<0.01*		
Sexual IPV (past month)	Intervention	56/386 (14.5%)	43/365 (11.8%)	0.70 (0.44, 1.11)	0.12	0.81 (0.43, 1.53)	0.52
	Control	70/393 (17.8%)	41/351 (11.7%)	0.62 (0.39, 0.98)	0.04*		
Reproductive Coercion (past month)	Intervention	52/244 (21.3%)	31/225 (13.8%)	0.55 (0.32, 0.95)	0.03*	1.24 (0.59, 2.63)	0.57
	Control	51/255 (20.0%)	33/217 (15.2%)	0.68 (0.40, 1.15)	0.16		
	Treatment type	Midline (T2) Mean (SD)	Endline (T3) Mean (SD)	beta (95% CI)	*p*-value	beta (95% CI)	*p*-value
Use of community resources^a^ (past 3 mos)	Intervention	*N* = 387, 0.61 (1.22)	*N* = 365, 0.27 (0.87)	-0.33 (-0.45, -0.21)	<0.01*	-0.13 (-0.32, 0.05)	0.15
	Control	*N* = 393, 0.47 (1.18)	*N* = 351, 0.27 (0.77)	-0.21 (-0.35, -0.07)	<0.01*		
Safety planning behaviors^a^ (past 3 mos)	Intervention	*N* = 387, 3.70 (3.11)	*N* = 365, 2.60 (2.85)	-1.08 (-1.38, -0.78)	<0.01*	-0.28 (-0.71, 0.16)	0.21
	Control	*N* = 393, 3.31 (3.03)	*N* = 351, 2.40 (2.64)	-0.83 (-1.15, -0.52)	<0.01*		
Quality of Life (mental)^a^ (past month)	Intervention	*N* = 386, 37.85 (8.30)	*N* = 363, 37.20 (7.69)	-0.50 (-1.48, 0.49)	0.32	-0.51 (-1.89, 0.88)	0.47
	Control	*N* = 392, 36.49 (7.63)	*N* = 350, 36.42 (7.65)	0.01 (-0.97, 1.00)	0.98		

^a^ Outcome variable is treated as a continuous variable, therefore regression coefficient beta and its 95% confidence interval are reported

* Denotes significant finding

## Discussion

In this RCT of women with recent experiences of IPV who sought healthcare in public health clinics within Mexico City, significant reductions in IPV and significant increases in safety planning, use of community resources, and mental quality of life were seen, regardless of exposure to control or treatment conditions. However, the nurse-delivered counselling sessions did not significantly improve levels of IPV, reproductive coercion, safety planning behaviors, use of community resources, and mental quality of life compared with women who were in the control arm. This was the first RCT, to our knowledge, to evaluate a health sector intervention to address IPV and other health outcomes within a LMIC, on such a large scale and outside of antenatal care settings. While the current trial is one of a limited number of trials of a nurse-delivered intervention response to IPV within a LMIC, the findings mirror primary care and/or nurse-delivered interventions in higher income settings [5, 9, 10, 25]. Importantly, this research demonstrates that it is possible to rigorously evaluate a health sector IPV intervention program with vulnerable populations in a LMIC setting within an urban mega-city.

More research is needed as to why the standard of care (control) yielded the same impacts as the enhanced nurse-delivered intervention. The participants in this trial consisted of a highly vulnerable population, namely lower income women with recent IPV experiences. Thus, exposure to even a low-dose intervention may have yielded improvements for both control and treatment participants. Similar reductions in both arms have been found in a previous screening trial in Canada [29] and with Mexican American women within the United States [25].

Qualitative interviews (data not shown) with control participants also suggest that the experiences of being asked about IPV within a healthcare setting by the survey, research staff and the nurses also triggered self-reflection and behavioral changes within this vulnerable population. Due to ethical reasons, a “pure” control arm was not included in this study, and the control arm’s standard of care was more than most women would typically receive within these clinics. In addition, while overall loss to follow-up was minimal, there was differential attrition between control and treatment groups, such that women in the control arm who dropped out of the study experienced more IPV. Thus, since these women were no longer in the 15-month follow-up, the effects of the intervention may be attenuated.

Trial findings indicate that the nurse-delivered intervention yielded statistically significant improvements in safety planning and mental quality of life at 3 months following baseline. These intervention effects, however, were not observed at T3. It may be that women in the intervention arm had an initial uptake in implementing safety planning behaviors that were one-time actions. For instance, safety planning behaviors, such as establishing a code with neighbors, memorizing important phone numbers, or packing an emergency bag, may have been implemented at a higher level by treatment women in comparison to control women. Then, once implemented, these safety planning behaviors were not repeated on an ongoing basis, and thus were not captured at T3, which focused on past year behaviors only. Another explanation for the lack of significant intervention effects on safety planning behaviors may be due to the context of Mexico City. Mexico City is an earthquake-prone area and thus all residents are in the routine habit of planning for emergencies [30]. Thus, the concept of safety planning may have resonated with both treatment and control women throughout the duration of the study, as was suggested by qualitative interviews (data not shown).

Short-term, but not long-term, significant treatment effects on improved mental quality of life may have been observed due to women’s initial feelings of support and validation upon receipt of the nurse-delivered intervention. However, these initial impacts at T2 may not have persisted due to lack of ongoing structural support outside of the nurse delivered intervention, as this intervention did not address social norms or community context, or seek to improve other support services for women experiencing violence. A 7-year, US-based prospective study of women who sought social services for IPV experiences yielded similar short- and long-term findings regarding mental health [31]. Other health sector-based RCTs in non-LMIC settings have shown mixed findings regarding improvements in mental health, including quality of life [5, 10, 32].

The findings of this randomized trial must be considered within the context of important limitations. In addition to the aforementioned limitations, the intervention was only delivered by select nurses and was not a system-wide intervention. While guidelines recommend moving more towards a system-wide approach to preventing and responding to IPV within the health sector, such an approach was not feasible in our study setting. It should also be noted that neither the researchers nor the clinic staff were blinded to their study condition. Thus, there may be a possibility of contamination, as control nurses may have provided more comprehensive counselling to participants beyond what the study protocol asked of them. The current study also remained in frequent contact and offered monetary compensation to all study participants as a means to reduce attrition. The attrition rate of this RCT was low; however, ongoing contact from the research team may have also promoted behavior change for participants in both control and treatment arms. Significant findings pertaining to safety planning and mental quality of life, furthermore, may be restricted to statistical significance only, as the clinical significance of such changes are currently unclear. Finally, behaviors may have been carried out but not captured by the survey items due to the time period of assessments. For instance, use of community resources was only assessed as “in the past 6 months”. This may have underestimated the impact of the intervention on these behaviors.

These limitations notwithstanding, strengths of this trial include a large sample size, successful randomization, low attrition, high fidelity to the intervention by the nurses, the ability to conduct this work in a complex setting, and long term follow-up. We also adhered to strict ethical guidelines for conducting research on IPV.

## Conclusions

These study findings do not lend support to utilizing an enhanced nurse delivered intervention over standard of care to reduce IPV. However, it should be noted that pre-post improvements in outcomes in both control and treatment arms do lend support to the idea that nurses, and more broadly, the health sector, can play a supportive role in assisting women with IPV experiences. Moreover, findings highlight that this nurse-delivered intervention has the potential to improve mental health and safety planning behaviors in the short term, but not in the longer term, although the extent to which statistically significant findings translate to clinically significant findings is currently not clear. Future work should consider examining interventions that involve healthcare provider responses bundled with other sectors (e.g., economic empowerment, policy, housing, social norms) to more holistically address the needs of women with IPV experiences within LMICs.

## Additional files

Additional file 1: Items used to assess IPV. (DOCX 14 kb)

Additional file 2: Table 2: Regression coefficients of adjusted models with and without treatment by time interaction terms (T1 and T3). (XLSX 11 kb)

Additional file 3: Table 3: Regression coefficients of adjusted models with and without treatment by time interaction terms (T1 and T2). (XLSX 9 kb)

Additional file 4: Regression coefficients of adjusted models with and without treatment by time interaction terms (T2 and T3, controlling for baseline measure at T1). (XLSX 11 kb)

## Abbreviations

IPV: intimate partner violence
LMICs: low- and middle-income countries
MoH: Ministry of Health
RCT: randomized controlled trial
