# Supplementary material for: A nurse-delivered, clinic-based intervention to address intimate partner violence among low-income women in Mexico City: findings from a cluster randomized controlled trial
Source: BMC Med. 2017 Jul 12;15:128. doi: 10.1186/s12916-017-0880-y (PMC5506677; doi:10.1186/s12916-017-0880-y)
Supplement: Supplementary file 1 — Items used to assess IPV. (DOCX 14 kb) [file 12916_2017_880_MOESM1_ESM.docx]

| Appendix 1: Items used to assess IPV* | |
| --- | --- |
| Physical Violence | During the last year, has your current boyfriend/partner/spouse pushed or shoved you or pulled your hair? |
|  | During the last year, has your current boyfriend/partner/spouse ever shaken or shoved you? |
|  | During the last year, has your current boyfriend/partner/spouse ever twisted your arm? |
|  | During the last year, has your current boyfriend/partner/spouse ever hit you with his fist or with something else that could hurt you? |
|  | During the last year, has your current boyfriend/partner/spouse ever kicked you, dragged you or beaten you up? |
|  | During the last year, has your current boyfriend/partner/spouse ever hit you with a domestic object? |
|  | During the last year, has your current boyfriend/partner/spouse ever intentionally burned you? |
|  | During the last year, has your current boyfriend/partner/spouse ever tried to choke or strangle you? |
|  | During the last year, has your current boyfriend/partner/spouse ever threatened to use or actually used a gun, knife or mother weapon against you? |
|  | During the last year, has your current boyfriend/partner/spouse ever thrown an object to you? |
| Sexual Violence | During the last year, has your current boyfriend/partner/spouse ever forced you to have sexual intercourse by physically threatening you, holding you down or hurting you in some way? |
|  | In the last year, did you ever have sexual intercourse because you were intimidated by him or afraid he would hurt you? |
|  | During the last year, has your current boyfriend/partner/spouse ever used physical force in order to have sexual intercourse with you |

* World Health Organization: **WHO multi-country study on women's health and domestic violence against women: summary report of initial results on prevalence, health outcomes, and women's responses**. In*.* Geneva: WHO; 2005.
